# Burnout among young physicians and its association with physicians’ wishes to leave: results of a survey in Saxony, Germany

**DOI:** 10.1186/s12995-016-0091-z

**Published:** 2016-01-22

**Authors:** Birte Pantenburg, Melanie Luppa, Hans-Helmut König, Steffi G. Riedel-Heller

**Affiliations:** Institute of Social Medicine, Occupational Health and Public Health, University of Leipzig, Philipp-Rosenthal-Str. 55, 04103 Leipzig, Germany; Department of Health Economics and Health Services Research, University Medical Center Hamburg-Eppendorf, Martinistr. 52, 20246 Hamburg, Germany

**Keywords:** Burnout, Maslach Burnout Inventory, Physician attrition, Physicians’ wishes to leave

## Abstract

**Background:**

Concerns about burnout, and its consequences, among German physicians are rising. However, data on burnout among German physicians are scarce. Also, a suspected association between burnout and German physicians’ wishes to leave remains to be studied. Therefore, the extent of burnout, and the association between burnout and wishes to leave clinical practice or to go abroad for clinical work was studied in a sample of young physicians in Saxony.

**Methods:**

In a cross-sectional survey, all physicians ≤40 years and registered with the State Chamber of Physicians of Saxony, Germany (*n* = 5956) received a paper-pencil questionnaire inquiring about socio-demographics, job satisfaction, and wishes to leave clinical practice or to go abroad for clinical work. Response rate was 40 % (*n* = 2357). Burnout was measured with the German version of the Maslach Burnout Inventory - Human Services Survey (MBI) consisting of the subscales *emotional exhaustion* (feeling emotionally drained), *depersonalization* (feelings of cynicsm) and *personal accomplishment* (feelings of personal achievement in job). Variables associated with burnout, and the association between burnout and wishes to leave were assessed in multivariate logistic regression analyses.

**Results:**

For *emotional exhaustion* participants reached a mean of 21.3 [standard deviation = 9.74], for *depersonalization* a mean of 9.9 [5.92], and for *personal accomplishment* a mean of 36.3 [6.77]. Men exhibited significantly higher depersonalization than women (11.3 [6.11] versus 9 [5.62], *p* < 0.001). Eleven percent of participants showed a high degree of burnout on all subscales, while 35 % did not show a high degree of burnout on any subscale. Confirming that one would become a physician again, and higher satisfaction with the components “work environment” and “humaneness”, were associated with a lower chance for a high degree of burnout on all subscales. Higher *emotional exhaustion* and lower *personal accomplishment* were associated with an increased chance of wishing to leave clinical practice. Higher *emotional exhaustion* and higher *depersonalization* were associated with an increased chance of wishing to go abroad for clinical work.

**Conclusions:**

Preventing physician burnout may not only benefit the affected individual. It may also benefit the health care system by potentially preventing physicians from leaving clinical practice or from going abroad for clinical work.

## Background

The issue of burnout is of high concern in the current debate in Germany [1, 2]. In Germany, sick leave due to burnout has increased from 0.7 days/100 insured live years in 2004 to 9.1 days/100 insured live years in 2011 [3]. In a study from the USA, physicians were more likely to have symptoms of burnout, as measured with the Maslach Burnout Inventory (MBI), than the working general population [4]. In a national sample of US physicians 46 % and among family physicians in 12 European countries nearly 65 % of participants showed signs of high burnout [4, 5].

There have been several studies of burnout among German physicians within specific specialties. However, they are scarce and employ various measuring instruments. One of the most widely used instruments is the Maslach Burnout Inventory (MBI). It consists of the three subscales *emotional exhaustion*, *depersonalization* and *personal accomplishment* [6]. *Emotional exhaustion* reflects “feelings of being emotionally overextended” [6]. *Depersonalization* relates to an “impersonal response towards recipients of one’s service” [6]. *Personal accomplishment* stands for “feelings of competence and successful achievement in one’s work with people” [6], which turns into the opposite in a burnout situation.

Instruments other than the MBI are also in use and results are therefore difficult to compare: Klein et al. [7] used the Copenhagen Burnout Inventory and estimated that 49 % of participating surgeons met the burnout criteria. Employing the same instrument, Heinke et al. [8] revealed that 40 % of anesthetists were at high risk for burnout. Using the MBI, Böhle et al. [9] estimated that of participating German urologists 40, 37, and 11 % showed high *emotional exhaustion*, high *depersonalization* and low *personal accomplishment*, respectively. With the same instrument, Pajonk et al. [10] estimated that 4 % of emergency physicians met the criteria of burnout and 11 % were at risk for developing burnout. In a study by Voltmer et al. [11] employing the Work-related Behavior and Experience Patterns questionnaire (AVEM), 22 % of physicians of various specialties in medical practice were at risk of burnout. Richter et al. [12] performed a study employing the MBI with hospital physicians of various specialties but did not categorize the results into “low”, “medium” and “high” symptoms of burnout. Larger German studies covering a broad range of medical specialties and including both physicians working in inpatient and outpatient settings are, to the best of our knowledge, missing.

Mental distress among physicians has been associated with, among others, substance abuse, depression and family conflicts [13]. In several studies using varying instruments to measure burnout, the consequences of physician burnout were investigated. Apart from the negative effect on an individual’s health, burnout may negatively impact the healthcare system as a whole by lowering quality of patient care [14], decreasing physician productivity [15], and leading to significant costs for the healthcare system due to early retirement and reduction in clinical hours [16].

Importantly, burnout among physicians has also been associated with increased rates of job turnover intentions [17, 18], and the intent to leave the medical profession [19].

In Germany, there is an ongoing debate about a potential shortage of physicians, among other reasons, due to physicians leaving clinical practice and/or going abroad for clinical work [20–23]. To the best of our knowledge, however, there are no studies from Germany investigating the association between burnout and physicians’ wishes to leave.

To address these gaps in knowledge, we aimed to assess the following questions within a study on wishes to leave among young physicians in Saxony, Germany:

1. To what extent are symptoms of burnout present among the study population?
2. Is there an association between burnout symptoms and physicians’ wishes to leave clinical practice or to go abroad for clinical work?

## Methods

### Study population and study design

The study was designed as a cross-sectional study. In a comprehensive survey, all physicians up to 40 years of age and registered with the State Chamber of Physicians of Saxony (SLÄK) (*n* = 5956) were sent a paper-pencil questionnaire. Registration with the SLÄK is obligatory for all licensed physicians practicing medicine in Saxony or, if not practicing medicine, having their main residence in Saxony. Two thousand three hundred fifty-seven questionnaires were returned from September 2012 to February 2013, equaling a response rate of 40 %. Compared to the population of physicians in Saxony, the sample contained more women (58 % versus 66 %, *p* < 0.001) and participants were slightly younger (33.2 years versus 32.9 years, *p* < 0.001). The letter accompanying the paper-pencil questionnaire stated that sending back the filled out questionnaire was regarded as giving consent to participating in the study. All questionnaires were anonymous and at no point could a filled out questionnaire be related to the respective respondent. The study was approved of by the Ethics committee of the University of Leipzig (Ethik-Kommission an der Medizinischen Fakultät der Universität Leipzig).

### Questionnaire and instruments

The anonymous paper-pencil questionnaire was developed based on a literature review, existing, mostly German-language, instruments, and self-generated questions: It contained questions on general socio-demographics [24, 25]; self-generated questions on wishes to leave clinical practice, and respective reasons (based on [20, 23, 24, 26]), and wishes to go abroad for clinical work, and respective reasons (based on [23, 24]) (English translation: “How much do you currently wish you could go abroad for clinical work?” and “How much do you currently wish you could leave clinical practice?”. Possible answers were: „not at all“ (1), “rather not (2), ”don’t know (3), “rather yes” (4), “absolutely” (5)); questions addressing job satisfaction, mainly based on the job satisfaction questionnaire developed by Bovier and Perneger [26], which was slightly modified by eliminating one question and adjusting the wording to fit the German context, while adding questions on working climate and career opportunities (based on [20]), job security and relationship with superiors (based on [27]) and a question on gender equality. Thus, to measure job satisfaction, participants were asked to rate their satisfaction with the job situation as a whole as well as on 20 aspects on a 5-point Likert scale ranging from 1 = very dissatisfied to 5 = very satisfied. Kaiser criterion of eigenvalues >1 was applied and items with factor loadings ≥0.5 were included. Factor analysis (principal component analysis) with promax rotation revealed 5 underlying factors: factor 1: “work environment” (intellectual stimulation at work, relationship with superiors, relationship and professional exchange with colleagues, work enjoyment, working atmosphere); factor 2: “work load“ (work load, time for family, friends and leisure activities, stress level at work); factor 3: “patient care“ (possibility to treat patients as you deem optimal, possibility to refer patients to specialists whenever you deem it necessary, quality of the medical care you provide); factor 4: “structural aspects” (career opportunities, current income, job security, equality of women and men) and factor 5: “humaneness” (relationship with non-medical staff, relationship with patients).

Symptoms of burnout were measured with the German version of the Maslach Burnout Inventory - Human Services Survey (MBI) [6]. The MBI’s three subscales were analysed separately as suggested by Maslach et al. [6]: *emotional exhaustion* (ranging from 0 to 54 points), *depersonalization* (ranging from 0 to 30 points) and *personal accomplishment* (ranging from 0 to 48 points). Higher scores on the subscales *emotional exhaustion* and *depersonalization* indicate a higher degree of burnout, while a higher score on the subscale *personal accomplishment* indicates a lower degree of burnout.

Means were calculated and subscales were categorized into “low”, “average”, and “high” degrees of burnout using the cut off values suggested by Maslach et al. [6]: For the subscale *emotional exhaustion* this translates into ≤18, 19–26, and ≥27 points, respectively; for the subscale *depersonalization* into ≤5, 6–9, and ≥10 points, respectively; and for the subscale *personal accomplishment* into ≥40, 39–34, and ≤33 points, respectively. Before employing it in the study, the questionnaire was pretested by physicians for comprehensibility and usability. The questionnaire is available from the authors upon request.

### Data analysis

Of the 2357 questionnaires returned, 9 questionnaires were excluded from the analysis: 3 persons were older than 40 years, 1 person worked abroad and 5 questionnaires were incomplete. For the purpose of studying burnout, only questionnaires of physicians working in patient care who fully completed the MBI were analysed (*n* = 1784). Means on MBI subscales were compared with t-test, and proportions were compared with χ^2^ -test.

In multivariate logistic regression analyses, variables associated with a high degree of burnout were assessed for each subscale. Participants’ age (in years), clinical work experience (in years), and satisfaction with the factors “work environment”, “work load”, “patient care”, “structural aspects”, and “humaneness” were introduced as continuous variables. Female sex (reference category [rc]: male), being in a relationship (rc: no relationship), having children (rc: no children), having German citizenship (rc: no German citizenship), being a specialist (rc: no specialization), would become a physician again (rc: would not become a physician again or indecisive), working full-time (rc: part-time), working in an urban setting (rc: rural setting), working in an inpatient setting (rc: outpatient setting), having a leading position/having their own practice (rc: non-leading position/employee in a practice) were dummy coded before introducing them into the regression models. Participants with missing values on variables were excluded from the analyses.

Multivariate logistic regression analyses were also performed to study the association between burnout symptoms and wishing to leave. Dependent variables were wishing to leave clinical practice and wishing to go abroad for clinical work. Scores on the subscales *emotional exhaustion*, *depersonalization* and *personal accomplishment*, as well as age, were introduced as continuous independent variables. Female sex (rc: male), being in a relationship (rc: no relationship), and having children (rc: no children) were dummy coded before introducing them into the regression models. Participants with missing values on variables were excluded from the analyses.

As not all participants fully completed all questions, the percentages in the results section correspond to the number of actual answers given. Data analysis was performed with Stata 12.0 for Windows.

## Results

### Characteristics of the study population

The characteristics of the study population are detailed in Table 1. Eighty-three percent (*n* = 1457) of the physicians worked in an inpatient setting and of these 85 % (*n* = 1222) worked in a hospital with more than 100 beds. Fifty percent (*n* = 138) of the physicians working in an outpatient setting worked as primary care physicians.

**Table 1:** Characteristics of the study population (*n* = 1784)

Female [% (n)]	61 (1086)
Age (years) [mean (SD; range)]	32.8 (4; 25 – 40)
German nationality [% (n)]	91 (1611)
In a relationship [% (n)]	84 (1489)
Children [% (n)]	52 (935)
Length of work experience (years) [mean (SD; range)]	5.6 (3.8; 0 – 17)
Specialist [% (n)]	38 (671)
Works full-time [% (n)]	84 (1501)
Works in an inpatient setting [% (n)]	83 (1457)

Percentages were calculated with the number of answers actually given and may differ from calculations based on a denominator without missing values. SD = standard deviation

### Degree of burnout

The degree of experienced burnout was assessed with the Maslach Burnout Inventory - Human Services Survey (MBI). The means of each subscale are presented in Table 2. Men reached a significantly higher score than women on the subscale *depersonalization* (mean: 11.3 [standard deviation: 6.11] versus 9 [5.62], p <0.001). Subscales were also categorized into low, middle and high degrees of burnout (Table 2). Again, distribution differed significantly between men and women on the subscale *depersonalization*, with a higher proportion of men showing a high degree of burnout.

**Table 2:** Burnout symptoms

	All (*n* = 1784)	Women (*n* = 1086)	Men (*n* = 698)	*p* value
	Mean [SD]	Mean [SD]	Mean [SD]	
Emotional exhaustion	21.3 [9.74]	21.5 [9.67]	20.9 [9.84]	ns
Depersonalization	9.9 [5.92]	9 [5.62]	11.3 [6.11]	<0.001
Personal accomplishment	36.3 [6.77]	36.1 [6.77]	36.5 [6.77]	ns
Degree of burnout	% (n)	% (n)	% (n)	
Emotional exhaustion				ns
Low	42 (741)	40 (437)	44 (304)	
Average	28 (505)	29 (312)	28 (193)	
High	30 (538)	31 (337)	29 (201)	
Depersonalization				<0.001
Low	25 (452)	29 (318)	19 (134)	
Average	27 (481)	29 (320)	23 (161)	
High	48 (851)	41 (448)	58 (403)	
Personal accomplishment				ns
Low	36 (641)	35 (377)	38 (264)	
Average	33 (591)	34 (364)	33 (227)	
High	31 (552)	32 (345)	30 (207)	

Means were calculated for each subscale of the MBI, and compared between women and men using the t-test. MBI subscales were categorized into low, average and high degree of burnout, and distribution compared between women and men using χ2 -test. SD = standard deviation, ns = not significant

### Extent of high degree of burnout

To assess the overall extent of burnout symptoms, the percentage of participants showing a high degree of burnout over all of the scales was analysed.

Eleven percent of participants showed a high degree of burnout on all subscales, while 34 % of participants did not show a high degree of burnout on any subscale (Table 3). A higher proportion of men (12 % of men versus 11 % of women, *p* < 0.01), of participants working full-time (12 % of participants working full-time versus 6 % of participants working part-time, *p* < 0.05), and of participants working in an inpatient setting (12 % of participants working in an inpatient versus 8 % of participants working in an outpatient setting, *p* = 0.001) showed a high degree of burnout on all subscales (Table 3).

**Table 3:** Extent of high degree of burnout

High degree on	All	Women	Men	Full-time	Part-time	Outpatient	Inpatient
	% (n)	% (n)	% (n)	% (n)	% (n)	% (n)	% (n)
No subscale	34 (611)	37 (407)	29 (204)	34 (506)	37 (105)	44 (129)	32 (467)
1 subscale	34 (600)	31 (342)	37 (258)	33 (499)	36 (101)	32 (94)	34 (497)
2 subscales	21 (378)	21 (223)	22 (155)	21 (318)	21 (60)	16 (48)	22 (323)
3 subscales	11 (195)	11 (114)	12 (81)	12 (178)	6 (17)	8 (23)	12 (170)
Total	100 (1784)	100 (1086)	100 (698)	100 (1501)	100 (283)	100 (294)	100 (1457)

The percentage of participants showing a high degree of burnout on none, one, two or all of the MBI subscales was calculated. Using χ2 -test results were compared between women and men (*p* < 0.01); participants working full- or part-time (*p* < 0.05); and participants working in an in- or outpatient setting (*p* = 0.001)

Finally, a higher proportion of participants wishing to leave showed a high degree of burnout on all subscales (14 % of participants wishing to go abroad for clinical work versus 10 % of participants not wishing to go abroad for clinical work; and 24 % of participants wishing to leave clinical practice versus 7 % of participants not wishing to leave clinical practice, *p* < 0.001) (Table 4).

**Table 4:** Extent of high degree of burnout

High degree on	Wish to go abroad		Wish to leave clinical practice	
	No	Yes	No	Yes
	% (n)	% (n)	% (n)	% (n)
No subscale	39 (473)	24 (130)	40 (542)	16 (67)
1 subscale	33 (400)	36 (193)	35 (474)	29 (119)
2 subscales	19 (231)	26 (143)	18 (251)	30 (125)
3 subscales	10 (117)	14 (75)	7 (93)	24 (100)
Total	101 (1221)	100 (541)	100 (1360)	99 (411)

The percentage of participants showing a high degree of burnout on none, one, two or all of the MBI subscales was calculated. Using the χ2-test, results were compared between participants wishing to go abroad for clinical work and those who do not (*p* < 0.001); and participants wishing to leave clinical practice and those who do not (*p* < 0.001). Percentages may not add up to 100 % due to rounding

### Parameters associated with a high degree of burnout

To identify parameters associated with a high degree of burnout, multivariate logistic regressions were performed. The results are detailed in Table 5. Confirming that one would become a physician again, as well as higher satisfaction with the components “work environment” and “humaneness”, were associated with a lower chance for a high degree of burnout on all subscales. Higher satisfaction with the component “work load” was associated with a lower chance for a high degree of burnout on the subscales *emotional exhaustion* and *depersonalization*. Women were less likely to exhibit a high degree of burnout on the subscale *depersonalization*.

**Table 5:** Variables associated with a high degree of burnout on the subscales *emotional exhaustion*, *depersonalization* and *personal accomplishment*

	Emotional exhaustion			Depersonalization			Personal accomplishment		
Independent variable	OR [SE]	CI 95 %	*p*	OR [SE]	CI 95 %	*p*	OR [SE]	CI 95 %	*p*
Female sex	1.21 [0.17]	0.92–1.59	0.165	0.48 [0.06]	0.38–0.60	<0.001	1.06 [0.14]	0.82–1.37	0.642
Age	1.01 [0.03]	0.95–1.08	0.730	0.95 [0.03]	0.90–1.01	0.079	1.02 [0.03]	0.96–1.08	0.608
In a relationship	0.89 [0.16]	0.63–1.26	0.507	0.98 [0.15]	0.72–1.32	0.871	0.82 [0.13]	0.59–1.13	0.218
Having children	0.83 [0.13]	0.62–1.13	0.240	1.09 [0.14]	0.84–1.41	0.511	1.11 [0.16]	0.84–1.47	0.474
German citizenship	0.67 [0.14]	0.44–1.02	0.060	0.69 [0.13]	0.48–1.01	0.056	0.67 [0.13]	0.45–0.98	0.041
Clinical work experience	0.99 [0.04]	0.92–1.07	0.786	1.02 [0.03]	0.95–1.08	0.600	0.96 [0.03]	0.90–1.03	0.271
Specialist qualification	0.77 [0.16]	0.51–1.16	0.214	0.85 [0.15]	0.60–1.20	0.356	1.27 [0.24]	0.87–1.85	0.214
Would become a physician again	0.55 [0.08]	0.42–0.72	<0.001	0.77 [0.10]	0.60–0.98	0.036	0.50 [0.06]	0.39–0.65	<0.001
Full-time employment	1.16 [0.23]	0.79–1.72	0.452	0.87 [0.14]	0.63–1.20	0.400	1.16 [0.21]	0.82–1.65	0.405
Urban setting	0.88 [0.13]	0.66–1.18	0.410	1.21 [0.16]	0.94–1.56	0.133	0.94 [0.13]	0.71–1.23	0.640
Inpatient setting	0.65 [0.13]	0.43–0.96	0.031	0.88 [0.14]	0.64–1.22	0.446	1.27 [0.24]	0.88–1.84	0.204
Leading position/own practice	1.35 [0.31]	0.87–2.11	0.180	1.29 [0.25]	0.89–1.88	0.181	0.55 [0.12]	0.36–0.85	0.008
Satisfaction with									
- work environment	0.54 [0.06]	0.43–0.67	<0.001	0.75 [0.07]	0.61 – 0.90	0.003	0.71 [0.07]	0.58–0.87	0.001
- work load	0.30 [0.03]	0.25–0.37	<0.001	0.79 [0.06]	0.68–0.91	0.002	0.97 [0.08]	0.82–1.14	0.712
- patient care	0.92 [0.10]	0.75–1.12	0.402	0.76 [0.07]	0.63 – 0.91	0.002	0.58 [0.06]	0.48–0.70	<0.001
- structural aspects	1.10 [0.11]	0.91–1.33	0.339	1.05 [0.09]	0.89–1.24	0.564	0.95 [0.09]	0.80–1.14	0.584
- humaneness	0.79 [0.09]	0.63–0.99	0.043	0.54 [0.06]	0.44–0.67	<0.001	0.52 [0.06]	0.42–0.65	<0.001

Multivariate logistic regression analysis was performed to determine variables associated with a high degree of burnout on the subscales *emotional exhaustion* (R^2^ = 0.2223), *depersonalization* (R^2^ = 0.0955) and *personal accomplishment* (R^2^ = 0.1324). Analysis was performed with the data of 1656 participants. OR = odds ratio; SE = standard error, CI 95 % = 95 % confidence interval

### Association between burnout symptoms and wishes to leave

A higher score on the subscale *emotional exhaustion* and a lower score on the subscale *personal accomplishment* were associated with an increased chance of wishing to leave clinical practice (Table 6). Higher scores on the subscales *emotional exhaustion* and *depersonalization* were associated with an increased chance of wishing to go abroad for clinical work. In contrast, female sex, being in a relationship and having children were associated with a lower chance of wishing to go abroad (Table 6).

**Table 6:** Association between burnout symptoms and physicians' wishes to leave

	Wish to go abroad for clinical work			Wish to leave clinical practice		
Independent variables	OR [SE]	CI 95 %	*p*	OR [SE]	CI 95 %	*p*
Female sex	0.70 [0.08]	0.56 – 0.87	0.002	1.09 [0.14]	0.84 – 1.42	0.500
Age	1.02 [0.02]	0.99 – 1.05	0.172	1.00 [0.02]	0.97 – 1.04	0.858
In a relationship	0.71 [0.10]	0.53 – 0.95	0.019	1.14 [0.20]	0.80 – 1.62	0.462
Having children	0.44 [0.06]	0.34 – 0.57	<0.001	1.21 [0.18]	0.90 – 1.61	0.200
Score *emotional exhaustion*	1.03 [0.01]	1.01 – 1.04	<0.001	1.09 [0.01]	1.07 – 1.10	<0.001
Score *depersonalization*	1.02 [0.01]	1.00 – 1.04	0.036	1.01 [0.01]	0.98 – 1.03	0.512
Score *personal accomplishment*	1.00 [0.01]	0.98 – 1.01	0.759	0.96 [0.01]	0.94 – 0.98	<0.001

Multivariate logistic regression analysis was performed to determine variables associated with the wish to go abroad for clinical work (R^2^ = 0.0614) and the wish to leave clinical practice (R^2^ = 0.1307). Analysis was performed with the data of 1748 and 1757 participants, respectively. OR = odds ratio; SE = standard error; CI 95 % = 95 % confidence interval

## Discussion

This study investigated the extent of burnout symptoms among a large sample of young physicians in Saxony, Germany. The achieved mean scores on the MBI subscales (*emotional exhaustion*: 21.3, *depersonalization*: 9.9, and *personal accomplishment*: 36.3) were similar to those detected among European family doctors (*emotional exhaustion*: 24, *depersonalization*: 7, and *personal accomplishment*: 37) and among hospital physicians in Hamburg, Germany (*emotional exhaustion*: 21.8, *depersonalization*: 9.7, and *personal accomplishment*: 34.1) [5, 12]. Likewise, in the present study, the percentage of participants showing high burnout symptoms on all subscales (approximately 11 %) was comparable to that among European family doctors (12 %) [5]. In both studies only a third of participants (34 and 35 %) did not show high symptoms of burnout on any of the subscales [5]. As several instruments are in use to measure burnout, estimates need to be interpreted and compared with caution across different studies. However, the above named studies all employed the MBI. Thus, the present sample of physicians in Saxony does not seem to present more symptoms of burnout than other physician samples. However, burnout does exist in this sample and it should be addressed.

In the present sample, a higher percentage of men exhibited high symptoms of burnout in bivariate analyses. Men also showed higher degrees of *depersonalization*, an “impersonal response towards recipients of one’s service” [6], widely described as cynicism. Consistently, in multivariate analysis, female sex was associated with a lower chance for *depersonalization*. A possible explanation may be that the German society expects less of men than of women to be empathic and to show emotions, but rather to be strong and not to show weakness. Thus, the social context may promote men to experience, but also to express, feelings of depersonalization. Similarly, in a meta-analysis by Purvanova et al. [28] including studies covering a range of professions, men showed higher degrees of *depersonalization*, whereas women showed higher *emotional exhaustion*. Purvanova and colleagues argue that women rather report *emotional exhaustion* as the main symptom of burnout, whereas men tend to present with increased levels of *depersonalization* as the main symptom of burnout [28].

In bivariate analysis, physicians who work in an inpatient setting exhibited more burnout. In multivariate analysis, however, inpatient work was associated with a lower chance for high *emotional exhaustion*. This is consistent with a meta-analysis of worldwide studies: Outpatient-based physicians showed a higher degree of *emotional exhaustion* [29]. In contrast, in a recent study from the USA, internal medicine hospitalists and outpatient general internists did not differ regarding *emotional exhaustion* and *depersonalization.* However, lower *personal accomplishment* was more common among hospitalists [30]. In cross-sectional studies, such as the present one, no conclusions can be drawn on causality. It is therefore both conceivable that the work setting may promote burnout, or that burned out individuals chose a certain work setting. The inconsistent findings across studies may be due to differing burnout-promoting and –preventing features of inpatient and outpatient work settings depending on the location and the respective health system.

Among a sample of French physicians, low quality of teamwork was associated with burnout and highly associated with intentions to leave the medical profession [19]. Likewise, in multivariate analysis, higher satisfaction with the aspects “work environment” (including the elements relationship with superiors, relationship and professional exchange with colleagues) and “humaneness” (including relationship with non-medical staff, relationship with patients) were associated with a lower chance for burnout on all subscales. The component “work environment” also comprised the element “intellectual stimulation at work”. Consistently, in a sample of Swiss physicians, lower exposure to continuing education was associated with a higher risk for *depersonalization* [31].

Long-term studies are needed to define predictors of burnout instead of mere associations. It is conceivable that burnout may prevent good teamwork and prevent from perceiving the work environment as intellectually stimulating. However, it would make even more sense if good teamwork and an intellectually stimulating work environment contributed to mental well-being, reduced work-related stress, and also to the prevention of burnout. Moreover, quality of teamwork and training *can* be addressed and improved by specific measures in an effort to improve the work environment and to prevent burnout – in contrast to the work setting, as in- and outpatient settings most likely will always be needed in a health care system.

While neither being in a relationship nor having children were associated with a high degree of burnout, higher satisfaction with the factor “work load” (comprising the elements time for family, friends and leisure activities, stress level at work) was associated with a lower chance for high *emotional exhaustion* and *depersonalization*. In the context of this study, qualitative aspects related to private life seem to be more important than quantitative facts. German hospital physicians exhibit a higher degree of “work interfering with family” conflicts when compared to the general public [32]. Work-family conflict has been identified as an important risk factor for burnout among physician populations in Germany [32, 33]. These data support study results from the USA and France revealing an association between work-life conflicts and burnout [19, 34]. A previous analysis among the same physician population revealed that difficulties in reconciling work and private life were an important reason for wishing to leave clinical practice [22]. These data confirm an earlier study among German physicians where a higher degree of “work interfering with family” conflict was positively correlated with intentions to quit the job [32].

There is an ongoing discussion in Germany about whether physicians may leave clinical practice or go abroad for clinical work [20–23]. Therefore, we investigated whether burnout may be associated with wishing to leave clinical practice or to go abroad for clinical work. In the setting of this study, higher symptoms of burnout were indeed associated with wishing to leave: Interestingly, higher *emotional exhaustion* and lower *personal accomplishment* were associated with the wish to leave clinical practice. Higher *emotional exhaustion* and higher *depersonalization* were associated with the wish to go abroad for clinical work.

*Emotional exhaustion*, the core symptom of burnout, was associated with both the wish to leave clinical practice and the wish to go abroad for clinical work. We can only speculate why, in the setting of this study, lower *personal accomplishment* was associated with the wish to leave clinical practice but higher *depersonalization* with the wish to go abroad. Perhaps participants scoring low on *personal accomplishment* rather focus on professional success and hope for better achievements by changing the occupational field. In contrast, participants scoring high on *depersonalization* may consider a change of health care systems to allow for more, or less, contact with patients, with the additional benefit of the adventure of going abroad. To elucidate this, further research would be warranted on burned out physicians’ expectations associated with leaving clinical practice or going abroad.

It should be taken into account that also factors not measured in the context of this study may contribute to the development of wishing to leave, such as available career opportunities, financial investments, etc. In the setting of this study, female sex, being in a relationship and having children were associated with a lower chance of wishing to go abroad. Participants with a partner and/or children may be more rooted in their current living situation in Germany and not consider moving abroad attractive due to the difficulties of finding a job for the partner and day care or school for the children.

The present results are consistent with a recent study among French physicians in which burnout increased the risk for intentions to leave the medical profession [19]. Similarly, studies among physicians in Spain and in China revealed that burnout might be associated with job turnover intentions (thoughts of leaving, looking for new jobs within a year, willing to accept other better job opportunities) [17, 18]. To the best of our knowledge, our study is the first revealing an association between symptoms of burnout and physicians’ wishing to leave the country and work abroad in clinical care.

These findings raise the question of how to prevent or to reduce burnout among physicians. Two recent randomized controlled trials from Spain and the USA indicate that mindfulness-based interventions in a group setting may decrease physician burnout at the individual level [35, 36]. These data are backed by a meta-analysis including randomized controlled trials and pre-post studies among physicians in Spain, Argentina, Norway, and the USA [37]. The authors conclude that “cognitive-, behavioral-, and mindfulness-based approaches” may be useful tools in decreasing physician burnout [37]. Reports on organizational approaches are scarce. A hospital in the USA attempted a “culture change” by switching the focus from physician burnout to physician wellness [38]. Measures included team building, creating a supportive work environment, and regular self-assessments with the MBI, along with providing sources of help [38]. Also in the USA, a primary care group rated physician well-being as important as quality of care [39]. The intervention targeted “influence over work environment”, efficiency, and “satisfaction with clinical and human aspects of patient care” [39]. During the study period, physicians’ *emotional exhaustion* decreased significantly [39].

It remains to be evaluated whether similar approaches may be effective in reducing or preventing burnout among physician populations in Germany. A recent systematic review by Seidler et al. points out that multidimensional approaches are needed as burnout is most likely multifactorial, and which work related factors are perceived as strain depends on the individual [40].

Preventing or reducing symptoms of burnout among physicians will not only improve individual health and decrease personal suffering. It may also be beneficial for the health care system as a whole in terms of lower costs, e.g. by avoiding costly treatment of burned-out physicians as well as avoiding early physician retirement and decreased physician productivity due to burnout [15, 16]. Preventing or reducing symptoms of burnout among physicians may also lead to better quality of care [14]. Importantly, preventing or reducing burnout may potentially also prevent physicians from leaving the (German) health care system.

### Limitations

Although we investigated a large sample of physicians, the results cannot be generalized to the population of physicians in Saxony: The sample is younger than the population and the proportion of women is larger in the sample. Due to the anonymised nature of the survey, non-responders could not be followed up and reasons for non-participation could not be elucidated. It is possible that only those participants who felt that the topics applied to them, and might exhibit higher symptoms of burnout, responded. Thus, prevalence of burnout would be overestimated. However, it is also conceivable that those physicians with higher burnout symptoms did not respond to the survey, which would lead to an underestimation of the prevalence of burnout. Importantly, participants could not be followed up and therefore it is not possible to trace whether wishes to leave were actually put into practice. Among family practitioners in England, intention to leave predicted actually leaving [41]. Among US physicians not all physicians put their intentions to leave into practice [42]. However, the percentage of physicians who actually left was higher among those who had intentions to quit clinical practice than those who did not have these intentions [42]. Thus, also for the sample of the present study, it is likely that at least some of the physicians wishing to leave will put these wishes into practice.

The study was a cross sectional study and no conclusions regarding causality can be drawn.

## Conclusions

The sample of physicians in the present study was not more burdened by burnout than previous physician samples. Still, the extent of burnout among these young physicians in Saxony is of concern. Higher satisfaction with the components “work environment” and “humaneness” were associated with a lower chance for burnout on all subscales. This may be a starting point for developing measures to reduce or prevent burnout in this study population. Several recent studies suggest that interventions on the individual or organizational level may improve symptoms of burnout among physicians. Future projects should concentrate on adapting preventive measures to the reality of physicians in Germany.

Furthermore, higher *emotional exhaustion* was associated with a higher probability for wishing to leave clinical practice or to go abroad for clinical work. While the link between burnout and intentions to quit the job or the profession has been established in previous studies, to the best of our knowledge, this is the first study to investigate the association between burnout and the wish to leave the country and practice medicine abroad. Future studies are warranted to evaluate burned out physicians’ expectations associated with leaving clinical practice or going abroad for clinical work.

Preventing or reducing burnout among physicians will not only improve individual health and decrease personal suffering. Importantly, preventing or reducing burnout may potentially also prevent the German health care system from losing physicians.
